# 

**Published:** 1888-07

**Authors:** 


### BOOK NOTICES.

ANNUAL OF THE UNIVERSAL MEDICAL SCIENCES.—Edited by Charles E. Sajous, M. D., and Seventy Associate Editors, and over two hundred collaborators in various capacities, 5 Vols., with ample illustrations. Philadelphia & London : F. A. Davis, Publisher.

This is a work of very great value to the profession of medicine and surgery. The object as stated in the preface, has been " To collate the progressive features of medical literature at large, and clinical data from countries in which no literature exists and to present the whole once a year in a continued form, prepared by writers of known ability." Judging from an inspection of the work before us we are able to testify to the pains-taking thoroughness with which the above objects have been carried out. Their scope and magnitude embrace all countries and every form of disorder that flesh is heir to, including admirable treatises on Anatomy, Physiology and Pathology. To the active practitioner who is ambitions to keep abreast of the times in the knowledge of his profession, this masterpiece of annuals will be found indispensable

All those who have directed or assisted in its compilation, are deserving of praise. The index alone shows great painstaking in its preparation, a feature of great importance in a work of this kind.

OUR LITTLE MEN AND WOMEN.—BABYLAND : If pleasant stories, useful lessons and instructive engravings, are a charm to the little folks who are first coming forward upon life's untried stage, those of the present generation have abundant reason to rejoice in what is being done for them by the enterprising publishers of the above entitled works ; but we don't want our little ones to cry for them from January to July any more. Send along every time. D. Lathrop Company, Boston, $1,00 a year.

THE CENTURY'S Siberian Articles give us much new light upon the extent, climatic differences, population and recourses of that hitherto unappreciated land. There is no flagging or falling off in its varied contents, which, if anything, are more interesting than ever. Its pictures of Ranch life are very realistic.

The July issue, in honor of the united celebration, at Gettysburg, on the 1st, 2d and 3d inst. of the survivors of the two armies in hostile array twenty-five years ago, will contain an article descriptive of previous reunions of the Blue and the Gray, and a poem by one of the Gray's entitled " The High Tide at Gettysburg,"

TEMPERANCE AND PROHIBITION is the title of a work published by G. H. Stockton, M. D., 1209 Broadway, Oakland, Cal., and for sale at $1.

This work is the best of the kind that has come under our notice, as it gives much valuable information upon the history and constituents of spirituous liquors and their effect upon the human system.

SOUTHLAND MAGAZINE, Clay Springs, Florida, $2.00 a year. This new and welcome visitor has just made its appearance upon our table. In tone, manner and make-up it is highly creditable to its projectors and the South, to the advantages of which it is fully alive.

HEALTH FRAGMENTS, OR STEPS TOWARD A TRUE LIFE.—By George H. Everett, M. D., New York, Published by the Author, 148 E. 20th st.

This is literally a manual of health, with practical rules and information for its preservation no less than its restoration when impared by disease. In scope, it embraces the whole range of health destroying habits which are only too prevalent among our well to do classes, with many valuable suggestions as to the air we breathe, the food we should eat and the open air exercise we should take, in order to meet our natural requirements, and keep body and mind fit for their best uses.

Better to hunt in fields for health unbought,
Than fee the doctor for a nauseous draught."

